# Effects of the cardiac cycle on carotid intima-media thickness in ELSA-Brasil baseline assessment

**DOI:** 10.1016/j.clinsp.2025.100744

**Published:** 2025-08-09

**Authors:** Yasmin C.G. Silva, Isabela M. Bensenor, Danilo P. Meireles, Alessandra C. Goulart, Paulo A. Lotufo, Itamar S. Santos

**Affiliations:** a Centro de Pesquisa Clínica e Epidemiológica do Hospital Universitário da Universidade de São Paulo, São Paulo, SP, Brazil; b Departamento de Clínica Médica da Faculdade de Medicina da Universidade de São Paulo, São Paulo, SP, Brazil; c Departamento de Epidemiologia da Faculdade de Saúde Pública da Universidade de São Paulo, São Paulo, SP, Brazil

**Keywords:** Intima-media thickness, Variability, Cardiovascular risk, Cardiovascular risk factors

## Abstract

**Background:**

It is unclear to what extent the cardiac cycle influences carotid intima-media thickness CIMT values, especially in individuals with major Cardiovascular Risk Factors (CVRF). The authors analyzed CIMT variability across the cardiac cycle using baseline data from the Brazilian Longitudinal Study of Adult Health (ELSA-Brasil).

**Methods:**

The authors analyzed data from 9546 ELSA-Brasil participants, aged 35 to 74 in six Brazilian cities. CIMT image acquisition was ECG-gated during three cardiac cycles (70 to 90 frames). The authors calculated the Coefficient of Variation (CV), range, and interquartile range to assess variability using frame-by-frame data. In addition, the authors stratified the sample according to the presence of five major CVRFs: hypertension, diabetes, dyslipidemia, smoking, and family history of premature CVD.

**Results:**

The mean age of the sample is 51.5 years (56% women). CIMT variability was higher in individuals with major CVRF, except for a family history of premature CVD. As expected, variability was higher in measurements of the near wall (CV: 5.8% and 4.9% for left and right CCA, respectively) than those of the far wall (CV: 2.1% for both CCA). A significant positive trend was observed between variability measurements and the number of major CVRFs (all *p* < 0.001).

**Discussion:**

The authors found a significant CIMT variability across the cardiac cycle in a very large sample. The magnitude of this phenomenon is more important among individuals with CVRFs. CIMT acquisition protocols must consider the effect of the cardiac cycle to improve risk estimation and reproducibility.

## Highlights

• The authors studied CIM variability across the cycle and its association with CVRFs.
• The authors analyzed video CIMT imaging from 9546 ELSA-Brasil participants.
• Median coefficients of variation were 2 % (far wall) to 5 %‒6 % (near wall).
• CIMT varied ≥0.1 mm during the cycle in approximately 25 % of the exams.
• Cardiac cycle had higher effects on CIMT in participants with CVRFs.

## Introduction

Carotid Intima-Media Thickness (CIMT) is a surrogate marker of Cardiovascular Diseases (CVD). It is frequently analyzed using B-mode Ultrasound (USG), a non-invasive, easily accessible, and radiation-free technique.^1^ Alterations in CIMT values may precede the formation of atherosclerotic plaques^2^ and are associated with the occurrence of CVD, such as acute myocardial infarction and stroke.^3^

Large cohorts have adopted CIMT measurements as proxies for the atherosclerotic process. However, acquisition and reading protocols vary widely across studies. Some of these differences include the selection of measurement site, transducer frequency, the acquisition of video (as opposed to static) images, ECG gating, and the level of computer assistance during the process.

These inequalities in acquisition and reading may impair efficient comparisons across different studies and populations. However, sparse data quantifies the influence of image protocol choices on CIMT measurements, and most data are derived from small studies in young populations. El Jalbout et al.^4^ studied 120 children aged 10 to 13 from the Quebec Adipose and Lifestyle Investigation in Youth study. Images were acquired using three different techniques: 1) B-mode ultrasound, 2) Radiofrequency echo tracking, and 3) Radiofrequency speckle probability distribution. Although all methods detected higher CIMT in children with high BMI (> 85^th^ percentile), they correlated weakly (intraclass correlation coefficient, 0.34), suggesting a significant influence of image acquisition protocols on CIMT results.

There is evidence that the cardiac cycle also influences CIMT measurements, with peak values occurring at end-diastole and decreasing during systole. This occurs due to changes in vascular compliance, compressibility, and physiological adjustments in blood pressure. Menees et al.^5^ analyzed ECG-gated CIMT data from 49 children aged 6 to 19 years to verify whether variations in CIMT during the cardiac cycle existed in this group. They found that CIMT measurements changed during the cardiac cycle, increasing during QRS activation in the ECG. Tierney et al.^6^ evaluated 184 subjects aged 11 to 29 with Kawasaki syndrome. They found that CIMT measurements were higher when systematically performed during the R-wave in ECG compared to a protocol in which the sonographers subjectively chose the best frame for measurement based on image resolution.

The Brazilian Longitudinal Study of Adult Health (ELSA-Brasil) is a large prospective cohort of middle-aged adults at recruitment (35 to 74 years-old). ELSA-Brasil included CIMT measurement at baseline using video recording for acquisition and computer-aided reading. ELSA-Brasil creates a favorable scenario to quantify the influence of the cardiac cycle on CIMT measurements in an adult sample, including the effects induced by major Cardiovascular Risk Factors (CVRF). This article aims to quantify CIMT variability across the cardiac cycle and its association with the presence of five major CVRF (hypertension, diabetes, dyslipidemia, smoking, and family history of premature CVD) using ELSA-Brasil CIMT data at baseline.

## Methods

### Study design

ELSA-Brasil is a cohort study with 15,105 civil servants from six Brazilian cities (São Paulo, Belo Horizonte, Porto Alegre, Salvador, Rio de Janeiro, and Vitória). The baseline assessment was carried out from August 2008 to December 2010. All active or retired employees of the six institutions, aged between 35 and 74 years, were eligible for the study. The study complies with the Declaration of Helsinki. Institutional board approvals from all centers were granted (approval number 659/06, May 19^th^, 2006), and all subjects signed an informed consent form.^7^

ELSA-Brasil baseline data were obtained using a comprehensive set of questionnaires and clinical, laboratory, and imaging assessments, including anthropometry, blood pressure (at rest and after postural change), electrocardiogram, fasting and post-load glucose levels, serum cholesterol, and carotid ultrasound. Blood and urine samples were also cryopreserved in liquid nitrogen for future analyses.^8^ ELSA-Brasil study was approved by the Institutional Review Board of all participating centers (CAAE 0016.1.198.000–06). This article complies with the STROBE guidelines.

### CIMT measurement protocol in ELSA-Brasil

The CIMT measurement protocol at ELSA-Brasil^9^ was applied in a standardized manner to all participating centers. A Toshiba device (Aplio XG™) with a 7.5 MHz linear transducer was used for acquisition. The CIMT was measured in a predefined 1 cm long segment on the distal wall of the common carotid, starting at 1 cm below the carotid bifurcation. The acquisition was performed over three cardiac cycles, together with the electrocardiographic recording. After the acquisition, all video recordings were sent to the reading center in the ELSA-Brasil investigation site in São Paulo. During reading, images were considered valid when the anatomical guides of the common carotid arteries, the interfaces between the lumen and the distal vessel wall, and the interfaces between the media and the adventitia layers of the distant vessel wall could be visualized.^10^ All measurements were made using MIA™ software. MIA™ automatically identifies the intima-lumen and media-adventitia interfaces in each video frame. These boundaries are validated (or corrected as necessary) by trained technicians, and subsequently, the software discretizes the ROI in each frame to determine the minimum, mean, and maximum CIMT values. This procedure for CIMT reading ensures accurate measurements, which resulted in inter-observer intraclass coefficients of 0.98 for both left and right CCAs in ELSA-Brasil.^11^^,^^12^ After identifying the Region Of Interest (ROI) for CIMT measurement, MIA software discretizes the ROI in each frame to determine minimum, mean, and maximum CIMT values. By protocol, ELSA-Brasil uses measurements from the far carotid wall as standard for CIMT determinations. In this article, CIMT corresponds to the mean ROI CIMT values. In some analyses presented in this article, the authors utilized both far- and near-wall measurements.

### CIMT variability measurements

Each carotid video recording is typically composed of 70 to 90 frames. Frame-by-frame CIMT values for each exam were used in the present analyses to determine CIMT variability during the cardiac cycle. Range corresponds to the difference between maximum and minimum CIMT values during the exam. CIMT Interquartile Range (IQR) corresponds to the difference between the 25th and 75th percentiles of CIMT values during the exam. The Coefficient of Variation (CV) corresponds to the standard deviation of CIMT values during the exam, divided by the average value of these CIMT values. The CV is a valuable marker as it translates variability proportionally to mean values. This allows fair comparisons, for example, between left and right CCA (as left CIMT values are typically higher than right CIMT values).

### Other variables

Sex, age, and race were self-reported at baseline. Race was self-reported according to the question used by the Brazilian National Census as White, Mixed, Black, Yellow, or Native race. Body Mass Index (BMI) was calculated as the weight (kg) divided by squared height (m^2^). Carotid-femoral Pulse-Wave Velocity (PWV) was assessed in ELSA-Brasil baseline as a marker of arterial stiffness.^10^^,^^13^ Briefly, participants were in a supine position, and blood pressure was measured using standard procedures. The distance from the sternal furcula to the right femoral pulse was measured with a metric tape. Carotid and femoral pulses were recorded during ten consecutive cardiac cycles with computer assistance. Crude PWV is calculated by dividing the distance from the sternal furcula to the femoral pulse by the delay between carotid and femoral pulses. Blood pressure-adjusted PWV is calculated using linear models that include the blood pressure measured at the beginning of the examination. Five CVRFs were analyzed in this study. (1) A family history of premature CVD was defined as the occurrence of cardiovascular disease in a first-degree relative before age 60; (2) Hypertension was defined as using medications to treat hypertension, systolic blood pressure ≥ 140 mmHg, or diastolic blood pressure ≥ 90 mmHg; (3) Diabetes was defined as a medical history of diabetes mellitus, using medications to treat diabetes mellitus, fasting serum glucose ≥ 26 mg/dL, HbA1c levels ≥ 6.5 %, or a 2 h oral glucose tolerance test ≥ 200 mg/dL; (4) Dyslipidemia was defined as using lipid-lowering medications or an LDL cholesterol level ≥ 130 mg/dL; and (5) Smoking was self-reported at baseline. Prior CVD was defined as a medical diagnosis of myocardial infarction, stroke, or angina pectoris. Due to the small subsample sizes, the authors merged the 4 CVRFs and 5 CVRFs groups in some analyses.

### Study sample

From 10,943 ELSA-Brasil participants with complete CIMT data, the authors excluded 614 (5.6 %) individuals with self-reported angina pectoris, myocardial infarction, stroke, or myocardial revascularization; 619 (5.7 %) individuals for whom frame-by-frame CIMT data could not be retrieved; 22 (0.2 %) participants with less than 50 frames in any of the carotid exams or with more than 10 % of excluded frames during MIA analyses, and 142 (1.3 %) with missing data on the overt cardiovascular diseases or any CVRFs. Therefore, the sample consists of 9546 ELSA-Brasil participants.

### Statistical analysis

For the study sample descriptive data, categorical variables are presented as counts and proportions, and continuous variables are presented as means and standard deviations. The authors studied the distribution of CIMT variability measurements, stratifying the sample according to the presence of hypertension, diabetes, dyslipidemia, smoking, and family history of premature CVD. For these analyses, participants who were exposed to past or current smoking were reclassified as a single smoking status level.

In all groups, the authors describe CIMT variability distribution (median [interquartile range]) according to the CCA side (left or right) and vessel wall (near or far). Comparison among groups with and without each major CVRF is performed using Kruskal-Wallis tests. The authors also present the same analyses, stratifying the sample according to the number of major CVRFs (0 to 5) and analyzing trends across groups using the Jonckheere-Terpstra test. In a post-hoc analysis, to explore a potential mechanistic explanation for the association between CIMT variability and the number of major CVRFs, the authors analyzed whether CIMT variability measurements were correlated with crude or blood pressure-adjusted PWV values.

The authors assessed how CIMT variability could influence cardiovascular risk estimates in two ways. First, the authors calculated the proportion of individuals for whom the CIMT variability range exceeded 0.1 mm. This cutoff was selected because it was previously associated with a 10 % to 15 % increase in the risk of cardiovascular events.^14^ The authors also estimated the number of participants subject to CIMT reclassification as normal or abnormal if the cardiac cycle was not considered during the assessment. The authors considered a cutoff for abnormal CIMT at the 75th percentile based on age- sex- and race-specific distributions.^1^^,^^9^ For each participant, the authors calculated intervals for CIMT values with an extension equal to the range values and centered at the mean CIMT for both the left and right CCA. Participants were classified as subject to reclassification if the cutoff for abnormal CIMT was included in far-wall intervals.

The authors built multiple linear regression models as post-hoc sensitivity analyses to further explore the association between the number of CVRFs and CIMT variability. Models were adjusted for age, sex, race, and BMI. These adjusted models were used to calculate adjusted means for each CIMT variability measurement, stratified by the number of CVRFs. All analyses were performed using R software 4.4.0. The significance level was set at 0.05.

## Results

Study sample characteristics are presented in Table 1. The mean age of the sample is 51.5 years, with 56 % of the participants being women. The frequencies of hypertension, diabetes, dyslipidemia, past or current smoking, and family history of premature cardiovascular disease were, respectively, 31.8 %, 14.0 %, 44.1 %, 42.6 %, and 15.0 %. Mean CIMT values were 0.60 (far) and 0.69 (near) right CCA walls and 0.61 (far) and 0.69 (near) left CCA walls. The mean number of frames in each record was 82.7 ± 12.5 and 82.6 ± 12.6 for left and right CCA, respectively.

**Table 1:** Study sample characteristics.

	Men (*n* = 4202)	Women (*n* = 5344)	All (= 9546)
**Age (years; mean ± SD)**	51.6 ± 9.2	51.4 ± 8.7	51.5 ± 8.9
**Race (%)**			
White	2330 (56.1 %)	2982 (56.4 %)	5312 (56.2 %)
Brown	1126 (27.1 %)	1236 (23.4 %)	2362 (25.0 %)
Black	553 (13.3 %)	853 (16.1 %)	1406 (14.9 %)
Other	144 (3.5 %)	220 (4.2 %)	364 (3.9 %)
**Educational level (%)**			
Up to incomplete high school	695 (16.5 %)	473 (8.9 %)	1168 (12.2 %)
High school	1439 (34.2 %)	1901 (35.6 %)	3340 (35.0 %)
College or above	2068 (49.2 %)	2970 (55.6 %)	5038 (52.8 %)
**Monthly income (%)**			
< USD1245	1139 (27.2 %)	1359 (25.5 %)	2498 (26.3 %)
USD1245–3319	1736 (41.5 %)	2481 (46.6 %)	4217 (44.4 %)
≥ USD3320	1309 (31.3 %)	1484 (27.9 %)	2793 (29.4 %)
**Smoking status (%)**			
Never	2147 (51.1 %)	3329 (62.3 %)	5476 (57.4 %)
Past	1446 (34.4 %)	1352 (25.3 %)	2798 (29.3 %)
Current	609 (14.5 %)	663 (12.4 %)	1272 (13.3 %)
**Hypertension (%)**	1526 (36.3 %)	1509 (28.2 %)	3035 (31.8 %)
**Diabetes (%)**	741 (17.6 %)	600 (11.2 %)	1341 (14.0 %)
**Dyslipidemia (%)**	1886 (44.9 %)	2320 (43.4 %)	4206 (44.1 %)
**Family history of CVD (%)**	554 (13.2 %)	875 (16.4 %)	1429 (15.0 %)

USD, US Dollars; CVD, Cardiovascular Disease.

Table 2 and Supplementary Table 1 present CIMT variability in the entire sample, expressed as medians and interquartile ranges, and as means and standard deviations, respectively. As expected, CIMT variability was higher in measurements of the near wall (median CV of 5.8 % and 4.9 % for the left and right CCA, respectively) than in the far wall (median CV of 2.1 % for both CCA). For the right and left far walls (the most common CIMT measurement sites), the median CIMT variability range was 0.06 mm. Notably, the 75th percentile for CIMT variability range in these sites was 0.10 mm, indicating that for 25 % of the sample, CIMT values changed across the cardiac cycle by more than this level. Additionally, the authors observed that a high proportion of exams could be classified as having either abnormal (≥ 75th percentile from age-, sex-, and race-specific distributions) or normal CIMT values if the cardiac cycle was not considered during the assessment. The authors found that 1872 (19.8 %) and 1916 (20.3 %) participants would be subject to left and right CIMT reclassification, respectively, given the observed range of CIMT values in their exams. In Supplementary Tables 2 to 6, the authors present the distributions of CIMT variability measurements according to the presence of hypertension, diabetes, dyslipidemia, past or current smoking, and family history of premature cardiovascular disease. Except for a family history of premature CVD, CIMT variability was higher in individuals with major CVRFs compared to those without them. This conclusion is consistent regardless of the CIMT assessment site or selected variability measurement. Regarding the family history of premature CVD, only the IQR of far-wall CIMT was significantly higher for both CCAs in individuals with this CVRF compared to those without it.

**Table 2:** CIMT variability measurements (median; [25th percentile – 75th percentile]) in the sample.

CIMT assessment		Variability measurement	All (*n* = 9546)
Right CCA	Far	CoV	0.021 [0.015‒0.032]
		Range	0.060 [0.040‒0.100]
		IQR	0.013 [0.010‒0.020]
	Near	CoV	0.058 [0.033‒0.118]
		Range	0.200 [0.110‒0.390]
		IQR	0.040 [0.025‒0.090]
Left CCA	Far	CoV	0.021 [0.015‒0.032]
		Range	0.060 [0.040‒0.100]
		IQR	0.015 [0.010‒0.023]
	Near	CoV	0.049 [0.028‒0.101]
		Range	0.160 [0.090‒0.330]
		IQR	0.040 [0.020‒0.073]

CIMT, Carotid Intima-Media Thickness; CCA, Common Carotid Artery; CoV, Coefficient of Variance; IQR, Interquartile Range.

Table 3 and Supplemental Table 7 present CIMT variability measurements according to the number of risk factors, expressed as medians and interquartile ranges, and as means and standard deviations, respectively. For all indexes and CIMT sites, the authors found a significant positive trend in the number of CVRFs and CIMT variability. To minimize the influence of other major determinants of CIMT values, the authors further explored the association between the number of CVRFs and CIMT, estimating marginal means using multiple linear regression models, adjusted by age, sex, race, and BMI. In these analyses, adjusted means for most CIMT variability measures were higher in participants with two or more CVRFs compared to the group with no CVRFs (Supplemental Table 8). As a post-hoc analysis, the authors analyzed whether CIMT variability was associated with arterial stiffness (measured by PWV) in the present sample. The authors found very weak positive correlations for these putative associations. Pearson’s coefficients for the correlation between crude PWV and CIMT variability measurements ranged from 0.017 to 0.069. For the correlation between blood pressure-adjusted PWV and CIMT variability, coefficients ranged from 0.014 to 0.113 (Supplementary Fig. 1).

**Table 3:** CIMT variability measurements (median; [25th percentile – 75th percentile]), according to the number of CVRFs.

CIMT assessment		Variability measurement	No CVRFs (*n* = 1959)	1 CVRF (*n* = 3280)	2 CVRFs (*n* = 2592)	3 CVRFs (*n* = 1284)	4 CVRFs (*n* = 390)	5 CVRFs (*n* = 41)	p
Right CCA	Far	CoV	0.020 [0.015‒0.029]	0.020 [0.015‒0.031]	0.021 [0.015‒0.034]	0.023 [0.015‒0.036]	0.025 [0.017‒0.041]	0.027 [0.018‒0.046]	**<0.001**
		Range	0.050 [0.030‒0.080]	0.050 [0.040‒0.090]	0.060 [0.040‒0.110]	0.070 [0.040‒0.120]	0.080 [0.050‒0.150]	0.090 [0.060‒0.180]	**<0.001**
		IQR	0.010 [0.010‒0.020]	0.010 [0.010‒0.020]	0.020 [0.010‒0.030]	0.020 [0.010‒0.030]	0.020 [0.010‒0.030]	0.020 [0.010‒0.040]	**<0.001**
	Near	CoV	0.055 [0.032‒0.110]	0.056 [0.032‒0.114]	0.060 [0.032‒0.121]	0.064 [0.035‒0.128]	0.072 [0.040‒0.150]	0.063 [0.036‒0.141]	**<0.001**
		Range	0.180 [0.100‒0.360]	0.190 [0.100‒0.370]	0.210 [0.110‒0.420]	0.240 [0.130‒0.430]	0.270 [0.150‒0.510]	0.290 [0.150‒0.440]	**<0.001**
		IQR	0.038 [0.020‒0.070]	0.040 [0.020‒0.080]	0.048 [0.030‒0.090]	0.050 [0.030‒0.108]	0.060 [0.030‒0.130]	0.058 [0.035‒0.130]	**<0.001**
Left CCA	Far	CoV	0.019 [0.014‒0.028]	0.020 [0.015‒0.031]	0.021 [0.015‒0.032]	0.024 [0.017‒0.038]	0.026 [0.018‒0.040]	0.030 [0.019‒0.051]	**<0.001**
		Range	0.050 [0.030‒0.080]	0.050 [0.040‒0.090]	0.060 [0.040‒0.100]	0.080 [0.050‒0.130]	0.090 [0.050‒0.140]	0.100 [0.060‒0.240]	**<0.001**
		IQR	0.010 [0.010‒0.020]	0.010 [0.010‒0.020]	0.020 [0.010‒0.030]	0.020 [0.010‒0.030]	0.020 [0.010‒0.035]	0.030 [0.020‒0.045]	**<0.001**
	Near	CoV	0.045 [0.027‒0.087]	0.047 [0.027‒0.094]	0.050 [0.029‒0.106]	0.056 [0.031‒0.122]	0.054 [0.030‒0.125]	0.077 [0.038‒0.183]	**<0.001**
		Range	0.140 [0.080‒0.280]	0.150 [0.090‒0.310]	0.170 [0.090‒0.350]	0.200 [0.110‒0.420]	0.210 [0.100‒0.390]	0.290 [0.160‒0.490]	**<0.001**
		IQR	0.030 [0.020‒0.060]	0.038 [0.020‒0.070]	0.040 [0.020‒0.080]	0.050 [0.030‒0.095]	0.050 [0.030‒0.110]	0.060 [0.030‒0.113]	**<0.001**

CVRF, Cardiovascular Risk Factor; CIMT, Carotid Intima-Media Thickness; CCA, Common Carotid Artery; CoV, Coefficient of Variance; IQR, Interquartile Range.

## Discussion

In a cohort of apparently healthy adult individuals, the median coefficient of variation in CIMT values during the cardiac cycle was from approximately 2 % (far wall) to 5 %‒6 % (near wall). CIMT variability range above 0.1 mm was common. CVRFs in this sample were associated with higher CIMT variability during the cardiac cycle. This was evident for hypertension, diabetes, dyslipidemia diagnoses, and smoking. Additionally, the number of CVRFs was significantly associated with higher CIMT variability. PWV values were not correlated with CIMT variability measurements, suggesting that arterial stiffness is not a major factor in explaining the effects of the cardiac cycle on CIMT values.

Previous observational studies show that a 0.1 mm increase in mean CIMT raises the risk of cardiovascular events by 10 % to 15 %.^14^ In the same direction, a systematic review of 119 clinical trials (> 100,000 subjects, most interventions targeting traditional CVRFs) by Willeit et al.^15^ showed that a 0.01 mm/year reduction in CIMT progression (due to the interventions in experimental design) was associated with a 9 % decrease in the risk of myocardial infarction, stroke, revascularization, or fatal CVD. These findings support the interpretation that the CIMT variability described in the present analyses is not negligible. It may impact the assessment of subclinical atherosclerosis by ultrasound and the estimation of overall cardiovascular risk. Range is the CIMT variability measurement that represents the observed difference in CIMT values that may occur at random, when the cardiac cycle is not considered during CIMT acquisition. Median range varied from 0.06 mm (far left and right CCA walls) to 0.2 mm (near right CCA). This means that for 50 % of the individuals, the potential error is equal to or greater than these values.

Far wall measurements are recommended due to better consistency.^1^ Near-wall measurements are more prone to artifacts and are more dependent on gain settings. The present data supports the adoption of far wall protocols, preferably. Considering far wall measurements, the data suggest that protocols that do not standardize image acquisition according to the cardiac cycle phase may lead to differences exceeding 0.1 mm in CIMT measurements for 25 % of individuals, potentially resulting in inaccurate estimation of CVD risk. Similarly, Menees et al.^5^ analyzed CIMT measurements in 49 children at various points in the cardiac cycle. They found that mean CIMT values obtained during the QRS complex (end-diastole) were significantly higher than those obtained in other phases (*p* = 0.01). However, the magnitude of the difference was small (0.01 mm). Although conflicting evidence exists,^16^ the present data suggest that CIMT variability in adults is higher than in children. Despite some differences in study designs, the range values from the present sample indicate that CIMT variability can reach 10 % of the mean CIMT values in the far wall and 20 % to 25 % of the mean CIMT values in the near wall.

The authors also found that the higher the number of CVRFs, the higher the CIMT variability. This finding is consistent with the study by Polak et al., which found that pulse pressure and LDL-cholesterol were significantly associated with the CIMT range across the cardiac cycle.^17^ This may explain why CIMT variability is proportionally higher in adults than in children. On the other hand, the higher CIMT variability in adults (especially those with established CVRFs) is even more critical, as the potential of CIMT for cardiovascular risk assessment is highest for individuals without overt CVD and intermediate pretest risk.^18^

CIMT value distributions vary according to age, sex, race, and CVRF profile among populations.^9^ Additionally, there is still doubt about the determinants of CIMT values,^19^^,^^20^ which may be influenced by phenotypes other than the atherosclerotic process.^21^ This scenario makes this measurement more challenging to compare among studies. This is amplified by wide heterogeneity in CIMT image acquisition and reading among studies, including head positioning, ROI location, how plaque presence influences the protocol (including how the new site is selected), and the number of measurements performed in each frame. This heterogeneity is explicit in Table 4.^17^^,^^22^, ^23^, ^24^, ^25^, ^26^ However, these sources of variation can be minimized through protocol standardization and computer-assisted reading. With strict protocol, Santos-Neto et al.^11^ found very high intra- and inter-observer intraclass correlation coefficients (0.97 to 0.98) for mean CIMT reading. This was also demonstrated by Tierney et al.,^6^ who described higher reproducibility using an ECG-based reading protocol (during peak R wave) compared to a subjective frame selection by the sonographer based on best image resolution.

**Table 4:** CIMT acquisition and reading in large cohort studies.

Study	Site	Video recording	ECG-gating	ROI location	Computer-assisted reading
Multi-Ethnic Study of Atherosclerosis	Right CCA	Yes	No	1 to 1.5 cm proximal to the right bulb	Yes
Atherosclerosis Risk in Communities	Right and left CCA, bulb and ICA	No	No	1 cm proximal to the CCA dilation, 1 cm proximal to the bifurcation; 1 cm into the ICA branch.	No
Framingham Offspring Study	Right and left CCA	Yes	Yes	Image centered below the bulb, with the edge of the bulb to the left of the image, in a plaque-free area.	Yes
Carotid Atherosclerosis Progression Study	Right and left CCA, bulb and ICA	Yes	Yes	CCA (2 to 6 cm proximal to the bifurcation), bulb (0 to 2 cm proximal to the bifurcation), and ICA (0 to 2 cm distal to the bifurcation)	Yes
Rotterdam Study	Right and left CCA	No	Yes	Most distal 1-cm long segment of the CCA	Yes
Northern Manhattan Prospective Cohort Study	Right and left CCA, bulb and ICA	No	No	12 sites combining near and far walls of right and left CCA, bulb and ICA	Yes
ELSA-Brasil	Right and left CCA	Yes	Yes	Segment, starting 1 cm before the carotid bulb and extending 1 cm proximally	Yes

CIMT, Carotid intima-Media Thickness; ROI, Region of Interest; CCA, Common Carotid Artery; ICA, Internal Carotid Artery.

The present study has strengths. Previous studies on CIMT variability recruited smaller samples compared to ELSA-Brasil, were restricted to selected clinical scenarios, and/or had no computational assistance for frame selection.^5^^,^^6^^,^^17^ The authors could address a very large database of CIMT measurements from the ELSA-Brasil study, a cohort of apparently healthy individuals. Considering left and right CCA acquisitions in all participants, this comprises more than 1.5 million individual frames, all of which were subject to computer-aided semi-automatic reading. The ELSA-Brasil protocol features strict standardization, yielding very high reproducibility indexes and enabling these analyses to isolate the influence of the cardiac cycle from other factors. Clinical and laboratory examinations confirmed CVRF statuses. The only exception is a family history of premature CVD. This is a possible explanation for why a family history of premature CVD had a weaker association with CIMT variability compared to other CVRFs in these analyses.

The present study must be interpreted in the context of its limitations as well. ELSA-Brasil is not a population-based study. Participants’ educational levels and income are higher than those of the entire country. However, the prevalence of CVRFs in ELSA-Brasil is close to that found in urban Brazilian population-based studies.^8^ Although caution is always advisable when generalizing study findings to different settings, the authors do not believe the present results are specific to or biased by the study sample. The present finding of higher CIMT variability in individuals with more CVRFs, even using CoV as the variability index, suggests that this phenomenon is not only due to higher mean CIMT values but also likely involves intima-media composition and its consequences for arterial wall dynamics during the cardiac cycle. However, cohort studies have limitations in addressing mechanistic hypotheses, which should be complemented using other designs. On the other hand, these findings support the conclusion that the heterogeneity observed among CIMT acquisition and reading protocols must be addressed, and the uncertainty surrounding the underlying mechanisms for CIMT variability does not diminish its importance in clinical studies. The authors agree it also raises other important research questions, such as how this standardization should be oriented. Future studies analyzing subsets of frames in each exam (systole/diastole, or at specific points guided by ECG tracing) and incorporating follow-up data can establish which protocols would be preferable for both reproducibility and accuracy for cardiovascular risk assessment. The ELSA-Brasil baseline assessment does not include flow-mediated dilation data, which could enhance the ability to address the role of endothelial dysfunction in CIMT variability. The authors did not perform sequential CIMT acquisitions at baseline, so more prolonged (hours or days) intra-individual variability was not assessed. According to the present results, CIMT variability is associated with major CVRFs. In the context of the article, this supports the conclusion that heterogeneities among CIMT protocols in addressing the cardiac cycle phases may yield significant differences in CIMT values, especially in the subgroup of individuals with these risk factors. The authors acknowledge that these findings also raise the interesting hypotheses that (1) CIMT variability may be associated with CVD risk and (2) CIMT variability may be used to refine CVD risk estimates. However, it was beyond the scope of the present article to address these new hypotheses. Finally, although the data indicate that CIMT variability during the cardiac cycle should be considered in CIMT acquisition protocols, it does not allow us to infer which point(s) of the cardiac cycle should be used for measurement. This must be established considering which protocols are more closely related to future CVD events. Longitudinal studies with video-based CIMT acquisition, such as ELSA-Brasil, may be the best approach to address this knowledge gap in the future.

## Conclusion

The authors found significant variability in CIMT values across the cardiac cycle in a very large sample. The magnitude of this phenomenon is particularly pronounced among individuals with CVRFs, and it affects the interpretation of results for a substantial proportion of individuals. CIMT assessment protocols should consider the effect of the cardiac cycle to enhance risk estimation and reproducibility, utilizing ECG-gated measurements or standardized averaging criteria across multiple frames. Future studies incorporating follow-up data and analyzing subsets of frames during the cardiac cycle may provide further insight into the optimal protocols for CIMT acquisition and reading, ultimately aiming for accurate CVD risk assessment.

## Authors’ contributions

Yasmin Silva: Conceptualization; formal analysis; investigation; data curation; writing-original draft; Isabela Bensenor: Conceptualization; resources; data curation; writing-review & editing; project administration; funding acquisition; Danilo Meireles: Validation; investigation; writing-review & editing; Alessandra Goulart: Validation; Data curation; writing-review & editing; project administration; Paulo Lotufo: Conceptualization; Resources; writing-review & editing; project administration; funding acquisition; Itamar Santos: Conceptualization; methodology; formal analysis; data curation; writing-original draft; project administration; supervision.

## Declaration of competing interest

The authors declare no conflicts of interest.

## Appendix Supplementary materials

Image, application 1
