# Supplementary material for: Effects of the cardiac cycle on carotid intima-media thickness in ELSA-Brasil baseline assessment
Source: Clinics (Sao Paulo). 2025 Aug 9;80:100744. doi: 10.1016/j.clinsp.2025.100744 (PMC12357139; doi:10.1016/j.clinsp.2025.100744)
Supplement: Supplementary file 1 [file mmc1.pdf]

CLINICS-D-25-00655_Supplementary Material

Supplemental Table 1 CIMT variability measurements (mean ± standard deviation) in the sample.

CIMT assessment		Variability measurement	All (n = 9,546)
Right CCA	Far	CoV	0.033 ± 0.058
		Range	0.090 ± 0.132
		IQR	0.022 ± 0.039
	Near	CoV	0.094 ± 0.102
		Range	0.307 ± 0.337
		IQR	0.074 ± 0.106
Left CCA	Far	CoV	0.032 ± 0.061
		Range	0.090 ± 0.133
		IQR	0.022 ± 0.034
	Near	CoV	0.083 ± 0.100
		Range	0.267 ± 0.315
		IQR	0.064 ± 0.083

CIMT, Carotid Intima-Media Thickness; CCA, Common Carotid Artery; CoV, Coefficient of Variance; IQR, Interquartile Range.

Supplemental Table 2 CIMT variability measurements (median; [25th percentile – 75th percentile]), according to hypertension status at baseline.

CIMT		Variability measurement	No hypertension (n = 6,511)	Hypertension (n = 3,035)	p
Right CCA	Far	CoV	0.020 [0.015–0.031]	0.023 [0.015–0.037]	**<0.001**
		Range	0.050 [0.040–0.090]	0.070 [0.040–0.120]	**<0.001**
		IQR	0.010 [0.010–0.020]	0.020 [0.010–0.030]	**<0.001**
	Near	CoV	0.055 [0.032–0.113]	0.067 [0.036–0.134]	**<0.001**
		Range	0.180 [0.100–0.370]	0.240 [0.130–0.450]	**<0.001**
		IQR	0.040 [0.020–0.080]	0.050 [0.030–0.110]	**<0.001**
Left CCA	Far	CoV	0.020 [0.014–0.030]	0.023 [0.016–0.037]	**<0.001**
		Range	0.050 [0.040–0.090]	0.070 [0.050–0.120]	**<0.001**
		IQR	0.010 [0.010–0.020]	0.020 [0.010–0.030]	**<0.001**
	Near	CoV	0.047 [0.027–0.094]	0.054 [0.030–0.115]	**<0.001**
		Range	0.150 [0.080–0.310]	0.190 [0.100–0.390]	**<0.001**
		IQR	0.035 [0.020–0.068]	0.043 [0.030–0.090]	**<0.001**

CIMT, Carotid Intima-Media Thickness; CCA, Common Carotid Artery; CoV, Coefficient of Variance; IQR, Interquartile Range.

Supplemental Table 3 CIMT variability measurements (median; [25th percentile – 75th percentile]), according to diabetes status at baseline.

**CIMT**		**Variability measurement**	**No diabetes (n = 8,205)**	**Diabetes (n = 1,341)**	**p**
Right CCA	Far	CoV	0.020 [0.015–0.032]	0.023 [0.016–0.038]	**<0.001**
		Range	0.060 [0.040–0.090]	0.070 [0.050–0.120]	**<0.001**
		IQR	0.010 [0.010–0.020]	0.020 [0.010–0.030]	**<0.001**
	Near	CoV	0.057 [0.032–0.116]	0.068 [0.036–0.139]	**<0.001**
		Range	0.200 [0.100–0.390]	0.250 [0.130–0.470]	**<0.001**
		IQR	0.040 [0.020–0.080]	0.050 [0.030–0.115]	**<0.001**
Left CCA	Far	CoV	0.020 [0.015–0.031]	0.025 [0.017–0.039]	**<0.001**
		Range	0.060 [0.040–0.090]	0.080 [0.050–0.130]	**<0.001**
		IQR	0.010 [0.010–0.020]	0.020 [0.010–0.035]	**<0.001**
	Near	CoV	0.047 [0.028–0.097]	0.059 [0.032–0.125]	**<0.001**
		Range	0.160 [0.090–0.320]	0.210 [0.110–0.420]	**<0.001**
		IQR	0.040 [0.020–0.070]	0.050 [0.030–0.100]	**<0.001**

CIMT, Carotid Intima-Media Thickness; CCA, Common Carotid Artery; CoV, Coefficient of Variance; IQR, Interquartile Range.

Supplemental Table 4 CIMT variability measurements (median; [25th percentile – 75th percentile]), according to dyslipidemia status at baseline.

**CIMT**		**Variability measurement**	**No dyslipidemia (n = 5,340)**	**Dyslipidemia (n = 4,206)**	**p**
Right CCA	Far	CoV	0.020 [0.015–0.031]	0.021 [0.015–0.034]	**0.002**
		Range	0.060 [0.040–0.090]	0.060 [0.040–0.110]	**<0.001**
		IQR	0.010 [0.010–0.020]	0.020 [0.010–0.030]	**<0.001**
	Near	CoV	0.057 [0.033–0.117]	0.060 [0.033–0.121]	0.322
		Range	0.200 [0.100–0.390]	0.210 [0.110–0.410]	**0.003**
		IQR	0.040 [0.020–0.080]	0.048 [0.030–0.090]	**<0.001**
Left CCA	Far	CoV	0.020 [0.015–0.031]	0.021 [0.015–0.033]	**<0.001**
		Range	0.060 [0.040–0.090]	0.060 [0.040–0.110]	**<0.001**
		IQR	0.010 [0.010–0.020]	0.020 [0.010–0.030]	**<0.001**
	Near	CoV	0.048 [0.028–0.097]	0.050 [0.029–0.107]	**0.022**
		Range	0.160 [0.090–0.320]	0.170 [0.090–0.350]	**<0.001**
		IQR	0.040 [0.020–0.070]	0.040 [0.020–0.080]	**<0.001**

CIMT, Carotid Intima-Media Thickness; CCA, Common Carotid Artery; CoV, Coefficient of Variance; IQR, Interquartile Range.

Supplemental Table 5 CIMT variability measurements (median; [25th percentile – 75th percentile]), according to smoking status at baseline.

**CIMT**		**Variability measurement**	**No smoking (n = 5,476)**	**Past or current smoking (n = 4,070)**	**p**
Right CCA	Far	CoV	0.020 [0.015–0.031]	0.021 [0.015–0.034]	**0.002**
		Range	0.060 [0.040–0.090]	0.060 [0.040–0.110]	**<0.001**
		IQR	0.010 [0.010–0.020]	0.020 [0.010–0.030]	**<0.001**
	Near	CoV	0.058 [0.032–0.117]	0.060 [0.033–0.122]	0.133
		Range	0.200 [0.100–0.390]	0.210 [0.110–0.410]	**<0.001**
		IQR	0.040 [0.020–0.080]	0.048 [0.030–0.090]	**<0.001**
Left CCA	Far	CoV	0.020 [0.015–0.031]	0.021 [0.015–0.033]	**<0.001**
		Range	0.060 [0.040–0.090]	0.060 [0.040–0.110]	**<0.001**
		IQR	0.010 [0.010–0.020]	0.020 [0.010–0.030]	**<0.001**
	Near	CoV	0.047 [0.028–0.098]	0.051 [0.029–0.106]	**0.006**
		Range	0.160 [0.090–0.320]	0.170 [0.090–0.360]	**<0.001**
		IQR	0.040 [0.020–0.070]	0.040 [0.020–0.080]	**<0.001**

CIMT, Carotid Intima-Media Thickness; CCA, Common Carotid Artery; CoV, Coefficient of Variance; IQR, Interquartile Range.

Supplemental Table 6 Variability measurements for CIMT values (median; [25th percentile – 75th percentile]), according to family history of premature CVD.

CIMT		Variability measurement	No family history of CVD (n = 8,117)	Family history of CVD (n = 1,429)	p
Right CCA	Far	CoV	0.021 [0.015–0.032]	0.021 [0.015–0.034]	0.552
		Range	0.060 [0.040–0.100]	0.060 [0.040–0.100]	0.232
		IQR	0.013 [0.010–0.020]	0.020 [0.010–0.028]	**0.032**
	Near	CoV	0.058 [0.033–0.118]	0.059 [0.034–0.119]	0.411
		Range	0.200 [0.110–0.390]	0.210 [0.110–0.400]	0.335
		IQR	0.040 [0.025–0.088]	0.048 [0.030–0.093]	**0.027**
Left CCA	Far	CoV	0.021 [0.015–0.032]	0.021 [0.015–0.033]	0.050
		Range	0.060 [0.040–0.100]	0.060 [0.040–0.100]	**0.007**
		IQR	0.015 [0.010–0.020]	0.020 [0.010–0.030]	**0.008**
	Near	CoV	0.049 [0.028–0.101]	0.049 [0.028–0.104]	0.807
		Range	0.160 [0.090–0.330]	0.170 [0.090–0.340]	0.343
		IQR	0.040 [0.020–0.070]	0.040 [0.020–0.080]	0.390

CIMT, Carotid Intima-Media Thickness; CCA, Common Carotid Artery; CoV, Coefficient of Variance; IQR, Interquartile Range.

Supplemental Table 7 CIMT variability measurements (mean ± standard deviation) according to the number of CVRFs

CIMT assessment		Variability measurement	No CVRFs (n = 1,959)	1 CVRF (n = 3,280)	2 CVRFs (n = 2,592)	3 CVRFs (n = 1,284)	4 CVRFs (n = 390)	5 CVRFs (n = 41)
Right CCA	Far	CoV	0.030 ± 0.053	0.032 ± 0.066	0.033 ± 0.054	0.035 ± 0.054	0.036 ± 0.038	0.040 ± 0.043
		Range	0.075 ± 0.121	0.083 ± 0.115	0.095 ± 0.130	0.109 ± 0.166	0.123 ± 0.177	0.135 ± 0.129
		IQR	0.019 ± 0.047	0.021 ± 0.038	0.024 ± 0.034	0.026 ± 0.035	0.031 ± 0.037	0.031 ± 0.028
	Near	CoV	0.087 ± 0.094	0.093 ± 0.106	0.094 ± 0.099	0.100 ± 0.104	0.111 ± 0.104	0.126 ± 0.175
		Range	0.272 ± 0.285	0.296 ± 0.339	0.316 ± 0.340	0.343 ± 0.366	0.377 ± 0.345	0.435 ± 0.761
		IQR	0.061 ± 0.080	0.071 ± 0.122	0.076 ± 0.088	0.089 ± 0.117	0.103 ± 0.126	0.110 ± 0.123
Left CCA	Far	CoV	0.031 ± 0.077	0.031 ± 0.063	0.030 ± 0.037	0.038 ± 0.069	0.036 ± 0.037	0.052 ± 0.085
		Range	0.071 ± 0.106	0.084 ± 0.127	0.091 ± 0.127	0.120 ± 0.184	0.122 ± 0.116	0.170 ± 0.159
		IQR	0.017 ± 0.025	0.021 ± 0.036	0.023 ± 0.029	0.029 ± 0.042	0.031 ± 0.043	0.052 ± 0.108
	Near	CoV	0.076 ± 0.091	0.080 ± 0.101	0.087 ± 0.109	0.090 ± 0.095	0.093 ± 0.099	0.117 ± 0.106
		Range	0.231 ± 0.282	0.251 ± 0.300	0.281 ± 0.327	0.313 ± 0.341	0.332 ± 0.378	0.399 ± 0.390
		IQR	0.053 ± 0.070	0.061 ± 0.089	0.069 ± 0.082	0.075 ± 0.081	0.079 ± 0.084	0.097 ± 0.097

CVRF, Cardiovascular Risk Factor; CIMT, Carotid Intima-Media Thickness; CCA, Common Carotid Artery; CoV, Coefficient of Variance; IQR, Interquartile Range.

Supplemental Table 8 Estimated marginal means for CIMT variability measurements (95% confidence intervals), from multiple linear regression models (adjusted for age, sex, race and body-mass index) according to the number of CVRFs

CIMT assessment		Variability measurement	No CVRFs (n = 1,946)	1 CVRF (n = 3,249)	2 CVRFs (n = 2,557)	3 CVRFs (n = 1,266)	4 + CVRFs (n = 426)
Right CCA	Far	CoV	0.040 [0.035–0.044]	0.041 [0.038–0.045]	0.042 [0.038–0.045]	0.043 [0.039–0.047]	0.044 [0.038–0.050]
		Range	0.116 [0.107–0.125]	0.120 [0.112–0.128]	**0.127 [0.119–0.135]**	**0.136 [0.127–0.145]**	**0.148 [0.134–0.162]**
		IQR	0.030 [0.028–0.033]	0.031 [0.028–0.033]	0.032 [0.030–0.035]	**0.034 [0.031–0.036]**	**0.037 [0.033–0.041]**
	Near	CoV	0.106 [0.099–0.114]	0.110 [0.103–0.116]	0.108 [0.101–0.114]	0.112 [0.105–0.119]	**0.120 [0.110–0.131]**
		Range	0.342 [0.318–0.365]	0.353 [0.332–0.373]	0.357 [0.336–0.377]	**0.373 [0.349–0.397]**	**0.398 [0.363–0.433]**
		IQR	0.087 [0.080–0.095]	0.092 [0.085–0.098]	0.092 [0.085–0.098]	**0.100 [0.093–0.108]**	**0.110 [0.099–0.121]**
Left CCA	Far	CoV	0.037 [0.033–0.041]	0.037 [0.033–0.041]	0.034 [0.031–0.038]	0.042 [0.037–0.046]	0.040 [0.034–0.047]
		Range	0.103 [0.094–0.112]	**0.111 [0.103–0.119]**	**0.112 [0.104–0.120]**	**0.135 [0.126–0.145]**	**0.138 [0.124–0.151]**
		IQR	0.025 [0.023–0.028]	**0.028 [0.026–0.030]**	**0.028 [0.026–0.030]**	**0.032 [0.030–0.035]**	**0.035 [0.031–0.038]**
	Near	CoV	0.090 [0.083–0.097]	0.092 [0.086–0.098]	**0.096 [0.090–0.103]**	**0.098 [0.091–0.105]**	0.099 [0.088–0.109]
		Range	0.307 [0.285–0.329]	0.316 [0.297–0.335]	**0.330 [0.311–0.349]**	**0.352 [0.330–0.374]**	**0.362 [0.329–0.395]**
		IQR	0.073 [0.067–0.078]	0.076 [0.071–0.081]	**0.079 [0.074–0.084]**	**0.083 [0.077–0.089]**	**0.084 [0.075–0.092]**

The authors excluded 105 participants (1.1%) due to missing data. $p<0.05$ for the comparison with the No CVRFs group is in bold.

CIMT, Carotid Intima-Media Thickness; CCA, Common Carotid Artery; CoV, Coefficient of Variance; IQR, Interquartile Range.

Supplementary Figure 1

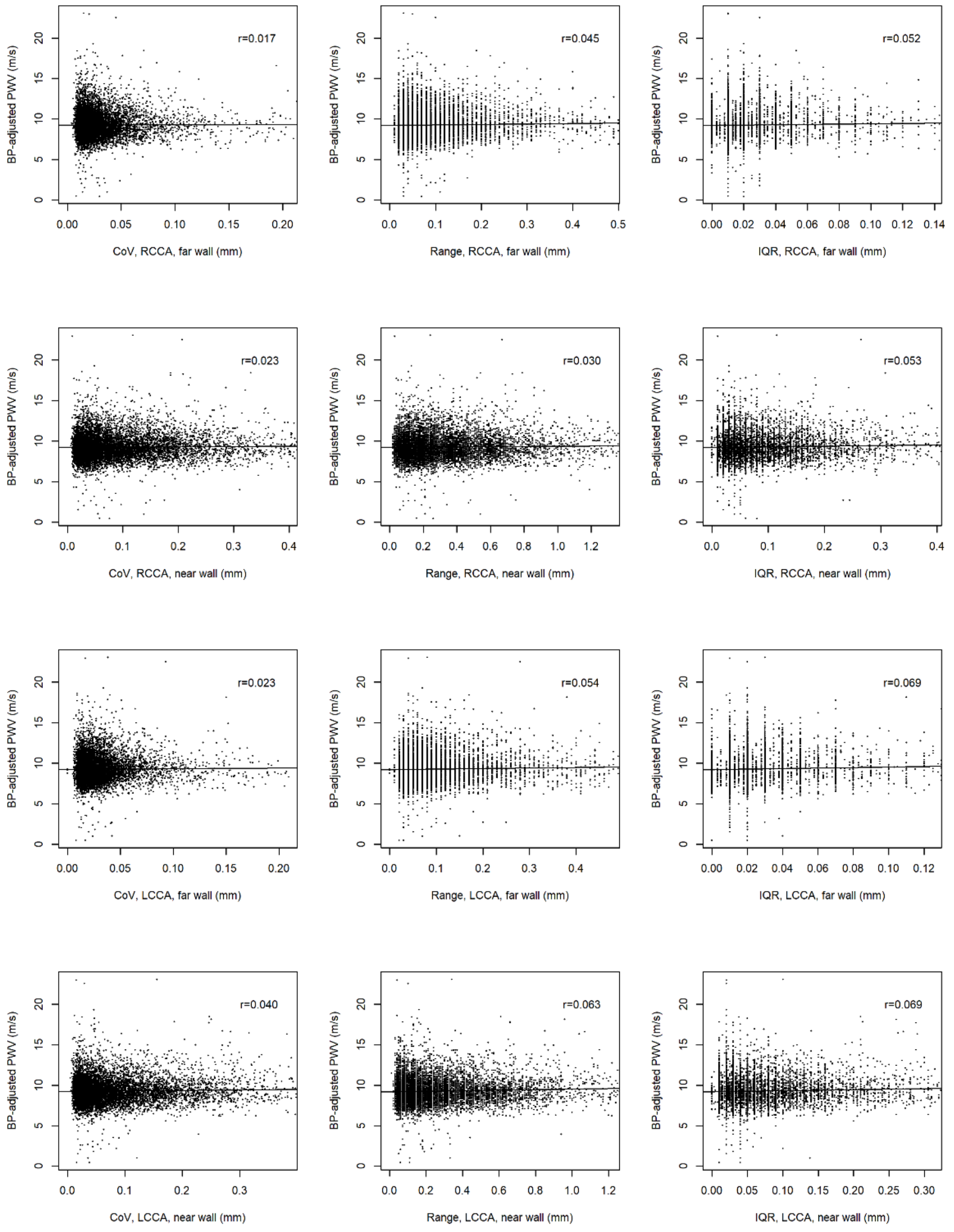

Fig 1: Dispersion plots and Pearson's correlations for CIMT variability measurements and blood-pressure adjusted PWV.
